# FOS and JUN regulate oxidative stress and steroidogenesis in human aldosterone-producing adenomas

**DOI:** 10.1016/j.redox.2025.103982

**Published:** 2025-12-16

**Authors:** Jia Wei, Eleonora Duregon, Mauro G. Papotti, Thomas Knösel, Martin Bidlingmaier, Silviu Sbiera, Martin Reincke, Tracy Ann Williams

**Affiliations:** a Medizinische Klinik und Poliklinik IV, Klinikum der Universität München, Ludwig-Maximilians-Universität (LMU) München, Munich, Germany; b Division of Pathology, Department of Oncology, University of Turin, Turin, Italy; c Institute of Pathology, Ludwig-Maximilians-Universität (LMU) München, Munich, Germany; d Department of Internal Medicine I, Division of Endocrinology and Diabetes, University Hospital, University Würzburg, Würzburg, Germany

**Keywords:** Adrenocortical adenoma, Primary aldosteronism, FOS, JUN, Oxidative stress, Steroidogenesis

## Abstract

Aldosterone-producing adenomas (APAs) are a major cause of primary aldosteronism (PA). While oxidative stress and steroidogenesis are intricately linked in adrenal disorders, their interplay and mechanistic basis in APA pathogenesis remain to be fully elucidated. Here, by integrating RNA sequencing of oxidative stress-exposed human adrenocortical cells with spatially-resolved transcriptomic profiling of human adrenal sections, we propose a previously unrecognized role for the activator protein-1 (AP-1) transcription factors FOS and JUN as key mediators linking oxidative stress to steroidogenesis in PA. Their expression and activation are spatially restricted, coinciding with regions of elevated oxidative stress. Phosphorylated FOS and JUN were exclusively detected in the adrenal cortex adjacent to functional adenomas (APAs and cortisol-producing adenomas), with negligible levels in cortex adjacent to non-functional adenomas and in normal adrenal cortex. In vitro, oxidative stress induced the upregulation and activation of FOS and JUN. Conversely, co-overexpression of FOS and JUN suppressed key steroidogenic genes (*StAR*, *CYP11B1*, *CYP11B2*), reduced aldosterone and cortisol secretion, and increased reactive oxygen species accumulation. Together, this work demonstrates that FOS and JUN may function in coordinating the redox-steroidogenesis axis, linking molecular changes in the adjacent cortex to tumor function and microenvironmental remodeling.

## Highlights

• FOS and JUN coordinate the redox-steroidogenesis axis.
• Oxidative stress drives spatial activation of FOS and JUN in peritumoral cortex of functional adrenal adenomas.
• Co-expression of FOS and JUN suppresses key steroidogenic genes and increases ROS.
• FOS and JUN couple redox imbalance with remodeling of adrenal tumor microenvironment.

## Graphical abstract

**Figure:**
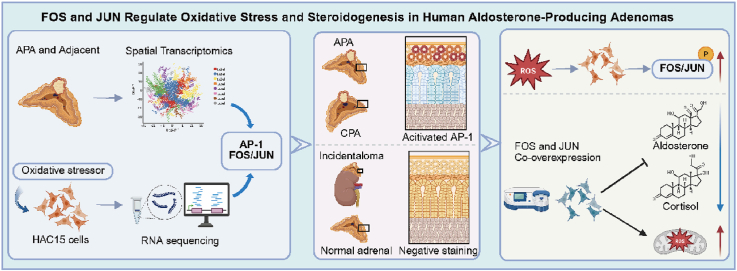

## 1 Introduction

Primary aldosteronism (PA) is the most common cause of endocrine hypertension, with an estimated prevalence of 5–15 % depending on hypertension severity, and up to 30 % in cases of resistant hypertension [1]. It is characterized by aldosterone overproduction from one or both adrenal glands, which is disproportionate to sodium intake and relatively independent of renin-angiotensin system regulation [2,3]. Aldosterone-producing adenomas (APAs) constitute a major cause of unilateral PA and are typically managed by adrenalectomy [4]. At the molecular level, APAs frequently harbor somatic mutations in genes encoding ion channels or transporters, predominantly in *KCNJ5*, *CACNA1D*, *ATP1A1* or *ATP2B3*, which trigger dysregulated aldosterone production [5]. Beyond these genetic lesions, emerging multi-omics studies have highlighted the contribution of the tumor microenvironment and cellular metabolism to adenoma function and growth [6,7]. Among these pathways, oxidative stress has emerged as a critical modulator of adrenal steroidogenesis [8,9]. This relationship appears bidirectional: steroidogenic activity inherently generates reactive oxygen species (ROS) as by-products of mitochondrial enzymatic processes, which promote lipid peroxidation in adrenocortical cells [9,10]. Conversely, ROS and oxidative stress can influence steroidogenic signaling pathways, either supporting normal hormone biosynthesis at physiological levels or inhibiting steroidogenesis under conditions of excessive oxidative insult. Thus, a dynamic feedback loop may exist whereby steroidogenesis and oxidative stress tightly regulate each other to maintain adrenal function and cellular homeostasis.

Our previous work demonstrated evidence of adaptation to oxidative stress in APAs [6,11], with oxidative stress markers such as malondialdehyde (MDA) and cyclooxygenase-2 (COX-2) being lower in APA tumor tissue compared with the surrounding non-neoplastic adrenal cortex [12]. This adaptive phenotype implies that APA cells may actively remodel their redox environment to support sustained steroidogenesis and growth. However, the transcriptional mediators that couple oxidative stress responses to altered aldosterone biosynthesis remain poorly defined. Identifying such regulators is crucial for understanding the molecular basis of redox adaptation in APAs and may reveal novel targets for therapeutic intervention.

To investigate the molecular mechanisms linking oxidative stress to altered steroidogenesis in PA, we modeled oxidative stress adaptation in the human adrenocortical cell line HAC15 using RSL-3 (a glutathione peroxidase 4 [GPX4] inhibitor) or hydrogen peroxide (H_2_O_2_). These treatments induce lipid peroxidation and excess ROS accumulation, thereby modeling some features of the oxidative microenvironment associated with PA. Through mRNA sequencing combined with integrative analysis of spatial transcriptomic datasets from APA-containing adrenal tissue sections, we identified *FOS* and *JUN* as genes of interest. As core components of the AP-1 transcription factor complex, FOS and JUN regulate cell proliferation, differentiation, death and, upon phosphorylation and activation, mediate cellular responses to oxidative stress [[13], [14], [15], [16]]. Additionally, AP-1 family members have been implicated in the regulation of steroidogenic acute regulatory protein (StAR) expression and steroid hormone synthesis in adrenocortical cells [[17], [18], [19], [20], [21]]. Based on these observations, we hypothesized that FOS and JUN act as key redox-responsive regulators linking oxidative stress adaptation to altered steroidogenesis in PA. Accordingly, we aimed to investigate their pathophysiological roles in human adrenal cells through integrative molecular profiling and functional assays, with the goal of elucidating how redox signaling intersects with transcriptional control of aldosterone production.

## 2 Materials and methods

### 2.1 Human adrenal tissue collection

Formalin-fixed paraffin-embedded (FFPE) and fresh frozen human adrenal tissues with APAs were obtained from the University Hospital LMU Munich, Germany. FFPE sections of cortisol-producing adenomas (CPAs) and adrenal incidentalomas were from the Division of Pathology, Department of Medical Sciences, University of Turin, Italy. Normal adrenal FFPE tissues were from nephrectomy patients at the Department of Internal Medicine I, Division of Endocrinology and Diabetes, University Hospital Würzburg, Germany. The study was conducted in accordance with local ethics committee guidelines and approved by the institutional review board (project numbers 379-10 and 24–0696). Written informed consent was obtained from all participants prior to sample collection.

### 2.2 Spatial transcriptomics data analysis

The spatial transcriptomics dataset (GSE274314) was retrieved from a previous study, with all samples originating from Munich [6]. Data were normalized and integrated in R using the Seurat package. To evaluate oxidative stress activity, module scores were calculated with the AddModuleScore function in Seurat based on the Hallmark Reactive Oxygen Species Pathway gene set from the Molecular Signatures Database (MSigDB), serving as a proxy for oxidative stress activity. These scores were visualized using integrated two-dimensional uniform manifold approximation and projection (UMAP) embedding and compared across groups using boxplots. Spatial feature plots were further generated to map oxidative stress scores onto H&E-stained tissue sections, thereby preserving the spatial context of oxidative stress activity.

### 2.3 Public transcriptomic datasets and integrative analysis

Three publicly available datasets (GSE156931 [22], GSE60042 [23], GSE64957 [24]) from Gene Expression Omnibus (GEO) database (https://www.ncbi.nlm.nih.gov/geo/), were incorporated for integrative analysis. Expression matrices were integrated, and batch effects were corrected using the ComBat function in the sva package. Differentially expressed genes (DEGs) between paired APA and adjacent adrenal tissue samples were subsequently identified using the limma package. Pathway activity was assessed using Gene Set Variation Analysis (GSVA) with the Hallmark gene sets from MSigDB (GSVA package). In addition, Gene Set Enrichment Analysis (GSEA) was performed with the ClusterProfiler package to further validate pathway-level alterations.

### 2.4 Next-generation sequencing and bioinformatics analysis

HAC15 cells were treated with vehicle control, 0.06 % dimethyl sulfoxide (DMSO; Sigma, D2650), 4 μM RSL3 (TOCRIS, 6118), or 250 μM H_2_O_2_ (Sigma, H1009) for 3 h before harvesting, total RNA extraction, reverse transcription and mRNA sequencing (Eurofins Genomics, Germany). In this study, all references to DMSO denote a concentration of 0.06 %, with solvent volumes equalized across all groups. The concentration of 4 μM RSL-3 was selected based on our previous research [12,25], while a concentration of 250 μM H_2_O_2_ was determined based on literature review [26] and preliminary observations of cellular responses.

Bioinformatics analyses were conducted in R using the DESeq2 package to normalize gene expression and identify DEGs, defined by an adjusted P-value <0.05 and |log2 fold change| > 1. Volcano plots were generated for visualization. Gene Ontology (GO) enrichment analysis of DEGs was performed using the ClusterProfiler package.

### 2.5 Cell culture

HAC15 human adrenocortical cells were cultured in DMEM/F12 medium (Gibco, 11330-032) supplemented with 10 % Cosmic Calf Serum (Hyclone, SH30087.03), 1 % ITS-A (Gibco, 5130044), and 1 % antibiotic-antimycotic solution (Gibco, 15240062). Cells were maintained at 37 °C in a humidified incubator with 5 % CO_2_. Routine mycoplasma testing using a PCR-based detection kit (ABM, G238) was conducted regularly, and all results were negative.

### 2.6 Establishment of HAC15 cells with Co-overexpression of FOS and JUN

One million HAC15 cells were co-transfected with *FOS* cDNA (GenScript, OHu22292) and *JUN* cDNA (Origene, SC118762) using the Cell Line Nucleofector™ Kit R (Amaxa, VCA-1001) according to the manufacturer's instruction. After 48 h of transfection, cells were harvested for PCR and Western blotting analysis.

### 2.7 Immunohistochemistry

Human adrenal tissue sections used for immunohistochemistry included both the adenoma and the surrounding non-neoplastic adrenal cortex, which is referred as the adjacent adrenal cortex. This region therefore comprises the cortex immediately surrounding the tumor as well as cortical areas not directly contiguous with the tumor but present within the same histological section.

FFPE sections (3 μm thick) were deparaffinized and rehydrated. Antigen retrieval was performed by heating the sections in Tris-EDTA buffer (pH 9.0) for 45 min. Endogenous peroxidase activity was blocked for 15 min, followed by incubation with blocking solution containing 5 % goat serum and 0.3 % Triton X-100 for 1 h at room temperature. Sections were then incubated overnight at 4 °C with primary antibodies against CYP11B2 (1:200, clone 17B; kindly provided by Prof. C.E. Gomez-Sanchez, University of Mississippi, USA; [27]) or FOS, JUN, P-FOS, and P-JUN (all 1:400; Cell Signaling Technology; product codes 2250, 9165, 5348, and 3270, respectively). Following three washes with Tris-buffered saline containing 0.05 % Tween 20 (TBST), sections were incubated with DAKO anti-mouse or anti-rabbit secondary antibodies for 1 h at room temperature. Immunoreactivity was detected using DAB chromogen (Agilent, K5007), followed by dehydration, clearing, and mounting with coverslips.

### 2.8 Cyto-immunofluorescence

Cells were washed three times with 0.05 % PBST (phosphate-buffered saline with 0.05 % Tween-20) and fixed with 4 % paraformaldehyde containing 0.3 % Triton X-100 for 20 min at room temperature. Cells were then blocked for 1 h at room temperature in PBS containing 5 % goat serum and 0.3 % Triton X-100, followed by overnight incubation at 4 °C with primary antibodies (FOS, JUN, P-FOS, or P-JUN) diluted 1:400. After three washes with PBST, cells were incubated for 1 h at room temperature in the dark with Alexa Fluor® 594-conjugated secondary antibody (1: 500, Cell Signaling Technology, 8889). Following three additional washes with PBST, cells were mounted using antifade mounting medium with DAPI (VECTASHIELD® HardSet™, H-1500-10). Images were captured using a Leica Stellaris 5 confocal microscope (Leica Microsystems, Wetzlar, Germany) and analyzed with ImageJ software (National Institutes of Health, USA). Immunofluorescence staining was quantified as the percentage of FOS-, JUN-, P–FOS–, or P-JUN-positive cells relative to DAPI-positive nuclei per field of view.

### 2.9 Quantitative real-time PCR analysis

Total RNA was extracted from HAC15 cells using the Maxwell RSC SimplyRNA Tissue Kit (Promega, AS1340) according to the manufacturer's protocol. Subsequently, 500 ng of RNA was reverse transcribed into cDNA (GoScript Reverse Transcription Kit, Promega, A2791). Real-time PCR amplification was performed on the BIO-RAD, CFX Duet PCR system using the following TaqMan probes from Thermo Fisher Scientific: *FOS* (Hs00170630_m1), *JUN* (Hs99999141_s1), *GAPDH* (Hs02786624_g1), *CYP11B2* (Hs01597732_m1), *CYP11B1* (Hs01596406_gH) or *StAR* (Hs00986559_g1). The expression levels of target genes were analyzed using the 2^−ΔΔCt^ method and normalized to the internal control gene *GAPDH*.

### 2.10 Western blotting

Cells were lysed on ice in RIPA buffer (Thermo Fisher Scientific, 89900) supplemented with protease inhibitor cocktail. Lysates were centrifuged at 14,000×*g* for 15 min at 4 °C, and protein concentrations in the supernatants were determined using a BCA assay kit (Thermo Fisher Scientific, 23227). Samples were denatured at 95 °C for 5 min in Laemmli sample buffer (Bio-Rad, 1610747), separated by SDS-PAGE, and transferred to 0.2 μm PVDF membranes using the Mini-Trans-Blot Cell system (Bio-Rad). Membranes were blocked for 1 h at room temperature in 0.1 % TBST containing 5 % non-fat dry milk, followed by overnight incubation at 4 °C with primary antibodies against FOS (1:1000, Cell Signaling Technology, 2250), JUN (1:1000, CST, 9165), and GAPDH (1:2500, CST, 2118). After three 10-min washes with TBST, membranes were incubated for 1 h at room temperature with horseradish peroxidase–conjugated anti-rabbit secondary antibody (1:2500, Cell Signaling Technology, 7074). Protein bands were visualized using enhanced chemiluminescence, and GAPDH was used as an internal loading control to normalize target protein expression.

### 2.11 Cell viability assay

Cell viability was assessed using the water-soluble tetrazolium salt-1 (WST-1) assay. HAC15 cells (2.5 × 10^4^ cells/well) were seeded in 96-well plates with starvation medium and incubated for 24 h. Cells were then treated with DMSO (Sigma, D2650), 4 μM RSL3 (TOCRIS, 6118), 4 μM RSL3 plus 10 μM Liproxstatin-1 (Lip-1; TOCRIS, 6113), or 250 μM H_2_O_2_ (Sigma, H1009) for 4 h. After addition of WST-1 solution (Roche, 11644807001), plates were incubated for 3 h at 37 °C in the dark. Absorbance was recorded at 450 nm and 690 nm using a FLUOstar Omega microplate reader (BMG LABTECH, Ortenberg, Germany). Cell viability was determined by subtracting the 690 nm absorbance from the 450 nm reading.

### 2.12 ROS assay

HAC15 cells (2.5 × 10^4^ cells/well) or transfected HAC15 cells (2.5 × 10^4^ cells/well) were seeded in 96-well plates with starvation medium and incubated for 24 h. HAC15 cells were then treated with DMSO, 4 μM RSL3, 4 μM RSL3 plus 10 μM Lip-1, or 250 μM H_2_O_2_ for 4 h, whereas transfected HAC15 cells were treated with either DMSO or 4 μM RSL3 for 4 h. Following treatment, all cells were incubated with 20 μM 2′,7′-dichlorofluorescin diacetate (DCFDA/H2DCFDA; Abcam, ab113851) in serum-free medium for 45 min at 37 °C in the dark to detect intracellular ROS. Fluorescence was immediately measured using a FLUOstar Omega microplate reader (BMG LABTECH, Ortenberg, Germany) at Ex/Em = 485/535 nm in end-point mode.

### 2.13 Aldosterone and cortisol assay

Cell culture supernatants were collected to quantify aldosterone and cortisol levels using the LIAISON® chemiluminescent immunoassay kit (DiaSorin Inc., Stillwater, MN, USA). Total protein concentrations of cell lysates were measured using a BCA protein assay kit (Thermo Fisher Scientific, 23227) to normalize aldosterone and cortisol levels.

### 2.14 Statistical analysis

The GraphPad Prism software (version 8.0; GraphPad, San Diego, CA, USA) was used for statistical analysis. Data normality was assessed using the Shapiro-Wilk test. All data were normally distributed, comparisons between two groups were conducted using Student's *t*-test, Welch's *t*-test, or paired *t*-test, as appropriate. For comparisons among multiple groups, one-way ANOVA with Dunnett's post-test was used, or Welch's ANOVA with Games-Howell's post-test in cases of unequal variances. Two-way ANOVA with Sidak's post-test was applied when analyzing two independent factors. Data are shown as the mean ± SEM, and statistical significance was considered as a two-tailed P < 0.05. *P < 0.05; **P < 0.01; ***P < 0.001; ns, not significant.

## 3 Results

### 3.1 Identification of oxidative stress-responsive genes *FOS* and *JUN* in human adrenocortical cells

**Fig. 1:**
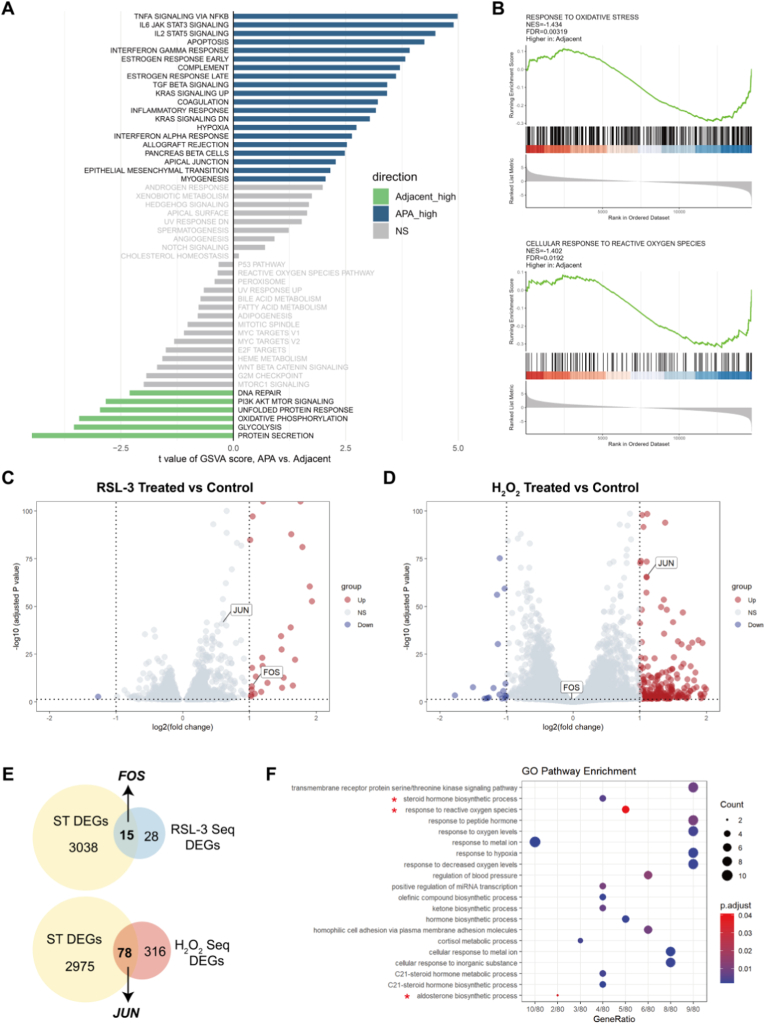
**Identification of oxidative stress-responsive genes *FOS* and *JUN* in human adrenocortical cells** **A**. Gene set variation analysis (GSVA) of hallmark pathways in APAs and adjacent tissues based on integrated datasets (GSE60042, GSE64957, and GSE156931). **B**. Oxidative stress-related pathways identified by gene set enrichment analysis (GSEA) of APAs versus adjacent tissues, based on integrated datasets (GSE60042, GSE64957, and GSE156931). **C** and **D**. Volcano plots of RNA sequencing data showing differentially expressed genes (DEGs) in HAC15 adrenocortical cells treated with 4 μM RSL-3 (oxidative stressor via GPX4 inhibition and lipid peroxidation) or 250 μM H_2_O_2_ (direct oxidant) for 3h vs. DMSO-treated controls (n = 3 biological replicates). Dashed lines: adjusted p < 0.05; |log2 fold change| > 1. **E**. Venn diagram identifying overlapping DEGs between RSL-3/H_2_O_2_-treated HAC15 cells and spatial transcriptomics (ST) data from aldosterone-producing adenomas (APAs, n = 7) vs. paired adjacent adrenal cortex. **F**. Gene Ontology (GO) enrichment of overlapping genes in Panel B, highlighting oxidative stress and steroidogenic pathways. NES, normalized enrichment score; FDR, false discovery rate; NS, not significant.

Based on integrated analyses of three publicly available GEO datasets (GSE60042, GSE64957, and GSE156931), GSVA revealed that the Hallmark oxidative phosphorylation pathway was significantly enriched in the adjacent adrenal tissues compared to APAs. Two oxidative stress-related pathways-the reactive oxygen species pathway and the peroxisome pathway-showed activity differences between APAs and adjacent adrenal tissues, although these did not reach statistical significance (Fig. 1A). To further evaluate this observation, we performed GSEA, which incorporates a broader gene related to oxidative stress. Both the response to oxidative stress pathway and the cellular response to reactive oxygen species pathway were significantly enriched in the adjacent adrenal cortex compared with APAs (Fig. 1B).

To functionally validate the involvement of oxidative stress in adrenocortical cells and to identify oxidative stress-responsive genes, HAC15 cells were exposed to 4 μM RSL-3, which induces oxidative stress via GPX4 inhibition and lipid peroxidation, or 250 μM H_2_O_2_, a direct oxidant generating exogenous ROS. RNA sequencing of HAC15 cells treated with RSL-3 versus DMSO controls identified 43 differentially expressed genes (DEGs; adjusted p < 0.05, |log2 fold change| > 1) enriched in lipid peroxidation pathways, while H_2_O_2_ treatment revealed 394 DEGs associated with acute oxidative stress responses (Fig. 1C and D). Cross-referencing these DEGs with spatial transcriptomic data from 7 APA-adrenal cortex pairs, we identified 15 overlapping genes within the RSL-3-associated lipid peroxidation signature and 78 overlapping genes from the H_2_O_2_ ROS response signature that were differentially expressed in APAs versus adjacent adrenal tissue (Fig. 1E). GO enrichment analysis of the overlapping gene sets implicated shared pathways in oxidative stress mitigation and steroid biosynthesis (Fig. 1F).

Although *FOS* and *JUN* were individually upregulated under different oxidative stress conditions, their potential cooperative function-given that they form the AP-1 transcription factor complex-may reflect shared or complementary cellular adaptive mechanisms to distinct oxidative stimuli. Therefore, *FOS* and *JUN* were selected as oxidative stress-related genes for further investigation.

### 3.2 Validation of *FOS* and *JUN* expression and oxidative stress activity in APAs and adjacent adrenal tissues

**Fig. 2:**
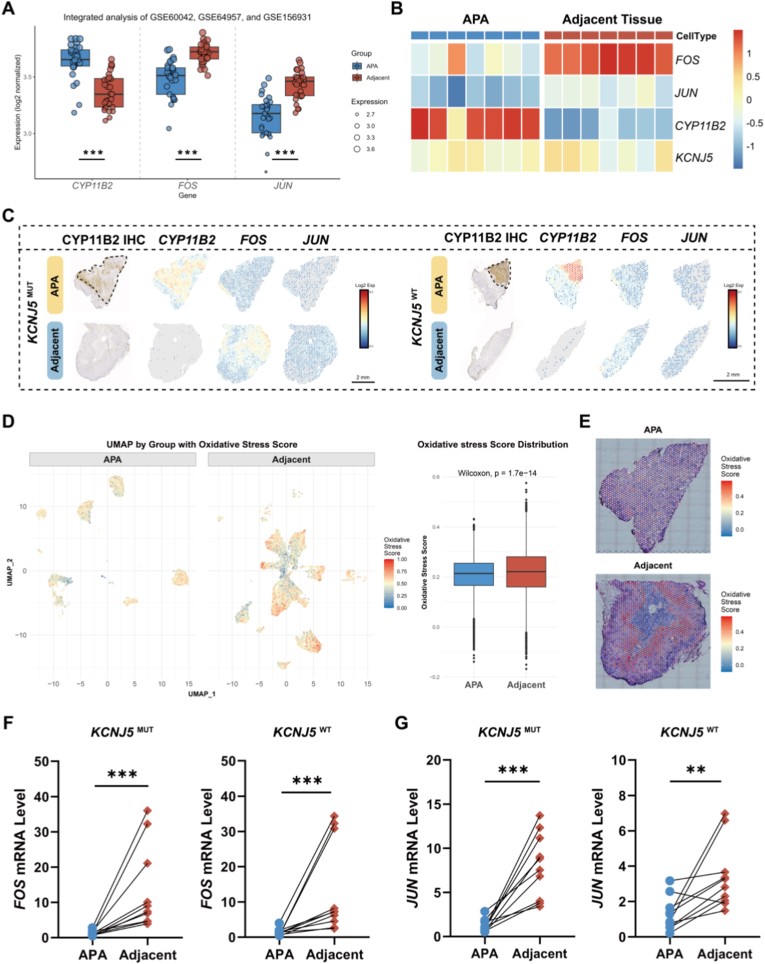
**Validation of *FOS* and *JUN* expression and oxidative stress activity in APAs and paired adjacent adrenal tissues** **A.** Boxplots showing the expression levels of *CYP11B2*, *FOS*, and *JUN* across APAs and paired adjacent adrenal tissues, integrating data from GSE60042, GSE64957, and GSE156931. **B**. Spatial transcriptomics heatmaps of *FOS, JUN, CYP11B2,* and *KCNJ5* across APA-adrenal cortex pairs (n = 7). **C**. Representative Spatial transcriptomics expression maps of *CYP11B2*, *FOS*, and *JUN* in *KCNJ5*-mutant (*KCNJ5*^MUT^) vs. wild-type APAs (*KCNJ5*^WT^) and adjacent tissues. **D**. Integrated UMAP embedding and boxplot showing the distribution of oxidative stress scores in seven APAs and their paired adjacent tissues. **E**. Representative spatial feature plots of oxidative stress scores projected onto H&E-stained APA and paired adjacent tissue sections. **F** and **G**. Gene expression analysis of *FOS* and *JUN* in APAs and paired adjacent adrenal tissues from 10 *KCNJ5*-mutant and 10 wild-type APA patients. *P < 0.05, **P < 0.01, ***P < 0.001.

Integrative analysis of three GEO datasets (GSE60042, GSE64957, and GSE156931) revealed lower *FOS* and *JUN* expression in APAs compared with paired adjacent adrenal tissues (Fig. 2A). Spatial transcriptomics across seven APA-adjacent pairs confirmed this pattern. *CYP11B2* and *FOS/JUN* exhibited mutually exclusive expression domains in both *KCNJ5*-mutant and wild-type tumors (Fig. 2B and C). Integrated UMAP embedding and boxplot of oxidative stress scores further showed higher oxidative stress activity in adjacent tissues than in APAs (Fig. 2D). Spatial feature plots mapped onto H&E-stained sections highlighted that elevated oxidative stress activity was localized predominantly to the adjacent adrenal cortex rather than the medulla (Fig. 2E). Real-time PCR analysis of cDNA from adrenal samples of patients undergoing surgery for PA demonstrated markedly lower *FOS* and *JUN* mRNA levels in APAs than in paired adjacent cortex, regardless of *KCNJ5* mutation status (Fig. 2F and G).

### 3.3 Differential expression and activation of FOS and JUN in adrenal tumors and adjacent adrenal cortex

**Fig. 3:**
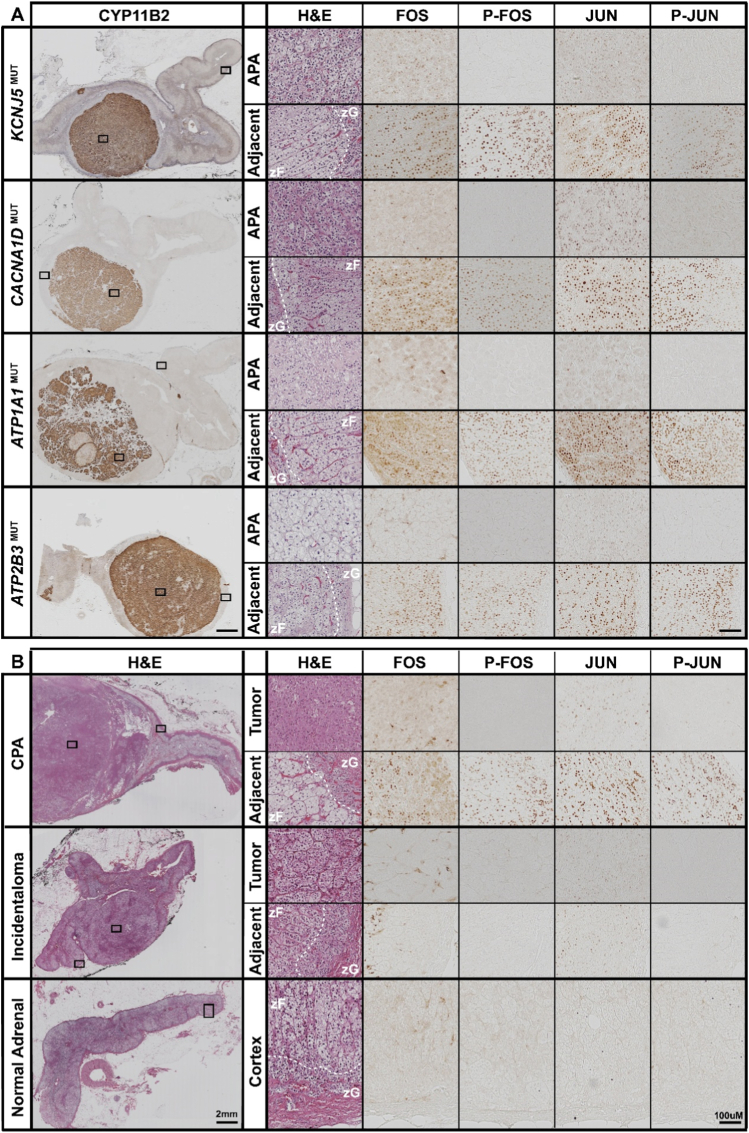
**Differential expression and activation of FOS and JUN in adrenal tumors and adjacent adrenal cortex** **A**. Immunohistochemistry of adrenal sections from APAs with mutations (MUT) in *KCNJ5*, *CACNA1D*, *ATP1A1* or *ATP2B3*. Sections were stained with hematoxylin and eosin (H&E) and immunostained for CYP11B2 (aldosterone synthase), total FOS, phospho-FOS (Ser 32; activated form), total JUN, and phospho-JUN (Ser 73; activated form). **B**. Immunohistochemistry of adrenal sections from cortisol-producing adenomas (CPAs), non-functional adrenal incidentalomas, and histologically normal adrenal cortex (from nephrectomy specimens). Sections were stained with hematoxylin and eosin (H&E) and immunostained for total FOS, phospho-FOS (Ser 32; activated form), total JUN, and phospho-JUN (Ser 73; activated form). Three biologically independent specimens were analyzed per group. zG: zona glomerulosa; zF: zona fasciculata.

We then performed immunohistochemistry to examine the expression and localization of FOS and JUN proteins and their phosphorylated forms, which represent their activated state, in adrenal tumors (APAs, CPAs, and non-functioning adrenal incidentalomas), as well as in the corresponding adjacent adrenal cortex and histologically normal adrenal tissue. Immunohistochemistry of APAs harboring mutations in any of the four main target genes (*KCNJ5, CACNA1D, ATP1A1* or *ATP2B3*) and their adjacent adrenal cortex consistently showed that total FOS and JUN proteins were expressed at higher levels in the adjacent cortex than within the APAs themselves. Notably, phosphorylated FOS and JUN were exclusively detected in the adjacent cortex and were absent in APAs (Fig. 3A). In CPAs, total FOS and JUN protein levels were markedly lower compared to the adjacent adrenal cortex, where atrophic changes were observed in zona fasciculata. Consistent with observations in APAs, the phosphorylated forms of FOS and JUN were largely absent in the tumor tissue but remained detectable in both zona glomerulosa and zona fasciculata cells of the non-neoplastic adjacent cortical regions. In contrast, both total and phosphorylated forms of FOS and JUN were undetectable in normal adrenal cortex, as well as in both the tumor and adjacent cortex of non-functioning adrenal incidentalomas (Fig. 3B).

### 3.4 Response of steroidogenesis and FOS/JUN expression to oxidative stress in human adrenocortical cells

**Fig. 4:**
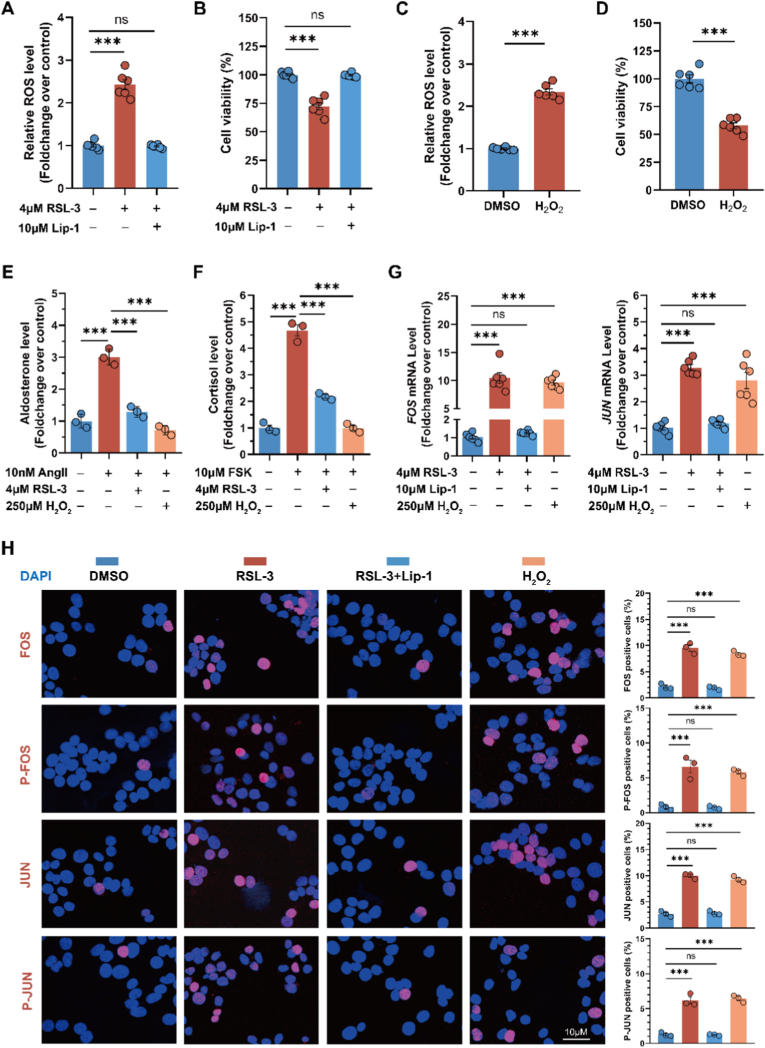
**Oxidative stress suppresses steroidogenesis and induces FOS and JUN expression and phosphorylation in human adrenocortical cells** **A** and **B**. Intracellular ROS level and cell viability in HAC15 cells treated with DMSO, 4 μM RSL-3 or 10 μM Lip-1 + 4 μM RSL-3 for 4h (n = 6 independent experiments). **C** and **D**. ROS level and cell viability in HAC15 cells treated with DMSO or 250 μM H2O2 for 4h (n = 6). **E.** Aldosterone secretion in HAC15 cells treated with DMSO, 10 nM Ang II, 10 nM Ang II + 4 μM RSL-3, or 10 nM Ang II + 250 μM H_2_O_2_ (n = 3). **F.** Cortisol production in HAC15 cells treated with DMSO, 10 μM FSK, 10 μM FSK +4 μM RSL-3, or 10 μM FSK +250 μM H_2_O_2_ (n = 3). **G**. Gene expression analysis of *FOS* and *JUN* in HAC15 cells treated with DMSO, 4 μM RSL-3, 10 μM Lip-1 + 4 μM RSL-3 or 250 μM H2O2 for 4h (n = 6). **H**. Cyto-immunofluorescence analysis of total FOS, phospho-FOS (Ser 32), total JUN, and phospho-JUN (Ser 73) in expression in HAC15 cells treated with DMSO, 4 μM RSL-3, 10 μM Lip-1 + 4 μM RSL-3 or 250 μM H2O2 for 4h (n = 3). Nuclei were counterstained with DAPI. Data in panels A–H were normalized to the DMSO control group. Statistical significance was determined by Welch's ANOVA with Games-Howell's post-test (panels A and B), Student's *t*-test (panels C and D), or one-way ANOVA with Dunnett's post-test (panels E–H). Data are shown as mean ± SEM; *p < 0.05; **p < 0.01; ***p < 0.001; ns, not significant. FSK: Forskolin.

To investigate the effects of oxidative stress on FOS and JUN expression, we stimulated HAC15 cells with 4 μM RSL-3 or 250 μM H_2_O_2_. Treatment with 4 μM RSL-3 for 4 h markedly increased intracellular ROS and reduced cell viability, both of which were effectively reversed by 1-h pre-treatment with 10 μM Lip-1 (Fig. 4A and B). Similarly, exposure to 250 μM H_2_O_2_ for 4 h significantly elevated ROS levels and reduced HAC15 cell viability (Fig. 4C and D). Notably, a shorter 3-h treatment with either RSL-3 or H_2_O_2_ failed to significantly alter ROS levels or cell viability, indicating that the oxidative stress response was not fully activated under these conditions. Correspondingly, RNA sequencing revealed no simultaneous upregulation of *FOS* and *JUN*, suggesting that their induction depends on both the intensity and duration of oxidative stress. Therefore, the 4-h treatment was adopted for subsequent experiments to ensure robust activation of the oxidative stress response.

We further evaluated the effects of oxidative stress on steroid hormone biosynthesis. Given the intrinsically low basal steroid output in HAC15 cells, cells were pre-stimulated with Ang II (10 nM) or forskolin (FSK, 10 μM) for 12 h, followed by a 4-h co-treatment with RSL-3 (4 μM) or H_2_O_2_ (250 μM) in the continued presence of the respective stimulus. Steroid measurements showed that oxidative stress significantly suppressed Ang II-induced aldosterone and FSK-induced cortisol secretion (Fig. 4E and F). Additionally, treatment with RSL-3 or H_2_O_2_ markedly upregulated FOS and JUN mRNA levels (Fig. 4G). Immunofluorescence analyses further confirmed that both oxidative stress inducers increased total FOS and JUN protein levels, along with enhanced phosphorylation (Fig. 4H).

### 3.5 FOS and JUN modulate steroidogenesis and oxidative stress responses in human adrenocortical cells

**Fig. 5:**
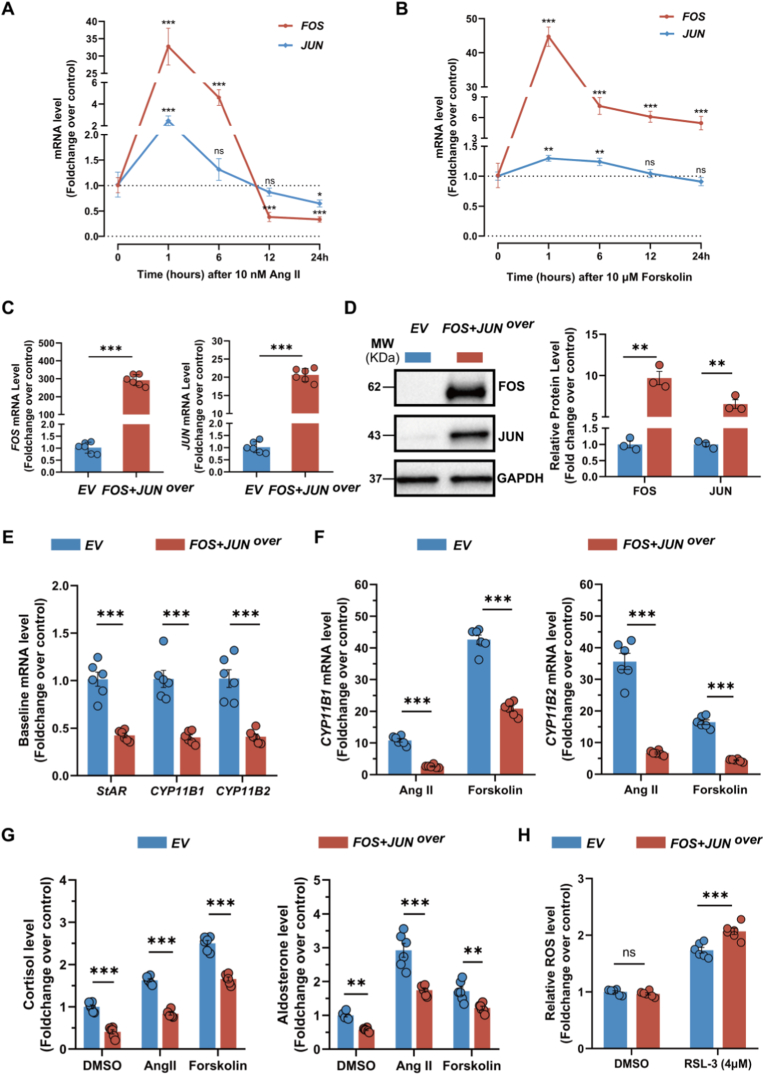
**FOS and JUN modulate the steroidogenic and oxidative stress response in human adrenocortical cells** **A** and **B**. Time-course analysis of *FOS* and *JUN* mRNA expression in HAC15 cells treated with 10 nM Ang II or 10 μM forskolin (n = 3 independent experiments). **C** and **D**. Gene (real-time PCR, n = 6) and protein (Western blot analysis, n = 3) expression of FOS and JUN in HAC15 cells after *FOS* and *JUN* co-transfection. **E**. Baseline expression of *StAR*, *CYP11B1* and *CYP11B2* in HAC15 cells with *FOS* and *JUN* co-overexpression (n = 6). **F**. *CYP11B1* and *CYP11B2* gene expression in HAC15 cells with *FOS* and *JUN* co-overexpression treated with 10 nM Ang II or 10 μM forskolin for 24 h (n = 6). **G**. Decrease in cortisol and aldosterone secretion in *FOS* and *JUN* co-overexpressing HAC15 cell supernatants treated with DMSO, 10 nM Ang II or 10 μM forskolin for 24 h (n = 6). **H**. ROS level in *FOS* and *JUN* co-overexpressing HAC15 cells treated with or without 4 μM RSL-3 for 4 h (n = 6). Data were normalized to the corresponding control group: **A and B** vs. DMSO, **C-E** vs. empty vector, **F–H** vs. empty vector (EV) + DMSO. Statistical significance was determined by one-way ANOVA with Dunnett's post-test relative to 0 h (panels A and B), Student's *t*-test (panels C and E), Welch's *t*-test (panel D) or two-way ANOVA with Sidak's post-test (panels F–H). Data are presented as mean ± SEM; *p < 0.05; **p < 0.01; ***p < 0.001; ns, not significant.

To investigate the potential roles of FOS and JUN in steroid hormone production, HAC15 cells were stimulated with 10 nM Angiotensin II (Ang II) or 10 μM forskolin to mimic pathological conditions associated with hyperaldosteronism and hypercortisolism. Time-course analysis revealed that Ang II induced a rapid and transient upregulation of *FOS* and *JUN* mRNA, followed by a gradual decline; notably, extended stimulation for 24 h suppressed their transcript levels below baseline (Fig. 5A). In contrast, short-term forskolin stimulation caused a sharp increase in *FOS* gene expression that later stabilized at a moderately elevated level, whereas *JUN* expression exhibited only minor fluctuations throughout the 24-h period (Fig. 5B).

To further explore the pathophysiological role of AP-1 in human adrenal cells, a co-overexpression model was generated by simultaneously transfecting HAC15 cells with plasmids encoding *FOS* and *JUN*. Real-time PCR showed marked overall upregulation of *FOS* and *JUN* transcripts in the co-transfected cells versus controls (Fig. 5C). Additionally, Western blot analysis demonstrated increased average FOS and JUN protein levels across the total cell population compared with empty vector controls (Fig. 5D).

Following model validation, both vector control and co-overexpression groups were treated with DMSO, 10 nM Ang II, or 10 μM forskolin. Co-overexpression of FOS and JUN significantly downregulated the expression of *StAR*, *CYP11B1* and *CYP11B2* under basal conditions (Fig. 5E), and this repressive effect on *CYP11B1* and *CYP11B2* persisted after Ang II or forskolin stimulation (Fig. 5F). Consistent with these transcriptional changes, cortisol and aldosterone concentrations in culture supernatants were significantly reduced in the co-overexpression group (Fig. 5G). Furthermore, while FOS and JUN co-overexpression did not affect basal intracellular ROS levels, it led to increased ROS accumulation in response to RSL-3 treatment compared to vector controls (Fig. 5H).

## 4 Discussion

This study identifies, to our knowledge for the first time, the AP-1 transcription factors FOS and JUN may act as key mediators coordinating the redox-steroidogenesis axis in human adrenocortical cells. We demonstrate that oxidative stress triggers phosphorylation and activation of FOS and JUN, which in turn orchestrate both redox adaptation and suppression of steroidogenic gene networks. Notably, FOS and JUN activation is restricted to the adrenal cortex adjacent to functional adenomas, highlighting a localized adaptive response to elevated ROS. These findings suggest that FOS and JUN link redox signaling to steroidogenic control, underscoring their critical role in maintaining redox homeostasis in the human adrenal cortex under conditions of oxidative stress.

The bidirectional interaction between AP-1 and oxidative stress encompasses both stress-induced activation of FOS and JUN [16,28] and redox-dependent AP-1 regulation by glutathione and thioredoxin systems [29,30]. In our in vitro model, sustained AP-1 overexpression paradoxically amplified ROS accumulation under oxidative challenge, indicating a critical threshold beyond which AP-1 activity transitions from cytoprotective to pro-oxidative. This duality may arise from adrenal-specific redox physiology, where high steroidogenic activity predisposes to elevated baseline ROS. Excessive ROS can impair mitochondrial function, thereby initiating a self-amplifying feedback loop that further elevates oxidative stress [31]. This ROS surplus drives lipid peroxidation, generating reactive aldehyde products such as 4-hydroxy-2-nonenal (4-HNE), which has been shown to enhance AP-1 expression and DNA-binding activity [32,33]. Sustained AP-1 activation, in turn, has been identified as a transcriptional suppressor of GPX4, a key antioxidant enzyme, thereby weakening cellular antioxidant buffering capacity and predisposing cells to ferroptosis [34]. Considering that hormone hypersecretion driven by functional adrenal adenomas further amplifies the requirement for redox-sensitive regulatory mechanisms, the shift of AP-1 from an adaptive to a pro-oxidative function is likely mediated by exhaustion of cellular antioxidant defenses and a finely tuned imbalance in intracellular redox status.

In the adjacent cortex of functional adenomas, steroidogenesis appears to be further suppressed by local oxidative stress characterized by excessive ROS, alongside systemic negative feedback on the hypothalamic-pituitary-adrenal (HPA) axis and the renin-angiotensin-aldosterone system (RAAS) due to dysregulated hormone secretion. Chronic oxidative stress activates MAPK pathways (JNK, ERK) [[35], [36], [37]], driving nuclear translocation of phosphorylated FOS and JUN [38,39] to repress steroidogenic genes (*StAR, CYP11B1, CYP11B2*). This AP-1-mediated repression may function as a mitochondrial protective mechanism, limiting steroidogenic flux and subsequent mitochondrial activity to prevent uncontrolled ROS production under sustained stress. In tissues without adenoma-related mitochondrial overload and ROS accumulation, such as normal adrenal cortex and non-functioning incidentalomas, MAPK-AP-1 signaling remains largely quiescent. In contrast, APA cells, despite persistent high steroidogenic activity and concomitant ROS generation, exhibit intrinsic redox adaptation characterized by upregulation of *GPX4* and related antioxidant systems [11]. This adaptive mechanism effectively buffers ROS, thereby restraining AP-1 activation, and allowing adenoma cells to bypass the protective “safety valve” of the surrounding cortex, thus sustaining pathological hormone hypersecretion.

Spatial transcriptomic profiling further revealed that oxidative stress activity in APAs was lower than in their surrounding cortex, consistent with previously reported immunohistochemical patterns of COX-2 and MDA [12]. Medullary regions adjacent to APAs, by contrast, display minimal oxidative stress, suggesting that this response is primarily confined to the cortex. Phosphorylated FOS and JUN, as key effectors of this response, are similarly restricted to the non-tumorous adrenal cortex. We propose they function to coordinate the local suppression of steroidogenic activity as a specific adaptive consequence of tumor-intrinsic redox adaptation and ROS accumulation in the adjacent cortical microenvironment.

Our in vitro findings in HAC15 adrenocortical cells replicated canonical steroidogenic responses to Ang II and forskolin [40]. Notably, we observed conserved FOS and JUN dynamics-transient induction by Ang II followed by suppression at 24 h-suggesting a feedback loop that constrains prolonged steroidogenesis under physiological conditions. Strikingly, co-overexpression of FOS and JUN dominantly repressed steroidogenesis even during hormonal stimulation, implying that loss of this AP-1-mediated repression in functional adenomas may permit pathological hormone hypersecretion. While primary cell validation is needed, this functional observation parallels the histopathological finding of phosphorylated FOS/JUN exclusion from tumor cells but retention in the adjacent cortex, encompassing both the zona glomerulosa and zona fasciculata, where suppression of steroidogenesis coincides with chronic oxidative stress signaling.

These findings also offer translational relevance for clinical practice. While CYP11B2 immunohistochemistry remains the standard for classifying APAs due to its specificity for aldosterone synthesis [27,41,42], CPAs lack comparably robust biomarkers and often rely on CYP11B1. However, this is an imperfect marker limited by nonspecific expression in non-neoplastic adrenal tissue and variable staining intensity within tumors. The spatially restricted expression of phosphorylated FOS and JUN in the adrenal cortex adjacent to functional adenomas introduces a novel ancillary criterion, particularly valuable for lesions with equivocal hormone secretion profiles. Consequently, these factors may serve as complementary tissue-based markers alongside CYP11B2 or CYP11B1, providing both enhanced diagnostic precision and insight into peritumoral oxidative stress dynamics.

Restoring AP-1-mediated transcriptional repression may represent a putative approach to mitigate hormone excess caused by adrenocortical tumors. However, systemic AP-1 activation risks off-target effects due to its pleiotropic roles in non-adrenal tissues. Encouragingly, adrenal-specific targeting opportunities may exist: tumor-suppressive roles of FOS [43,44] and JNK-phosphorylated JUN [45], alongside evidence of MAPK pathway hyperactivity in functional adenomas [[35], [36], [37]], suggest that pharmacological inhibition of JNK/ERK signaling (e.g., via small-molecule inhibitors) could provide a means to selectively normalize AP-1 activity in diseased tissue, potentially reversing hormone excess.

Future studies should leverage single-cell RNA sequencing and high-definition spatial transcriptomics to dissect the cellular and spatial heterogeneity of AP-1 activation, oxidative stress responses, and steroidogenic regulation in APAs and their surrounding cortex. Furthermore, complementary immunohistochemical validation in larger retrospective adrenal cohorts could definitively confirm the spatial restriction of phosphorylated FOS and JUN, bridging mechanistic insights with tissue-level observations. Building on these insights, preclinical testing of MAPK inhibitors in APA organoids or xenografts could assess the feasibility of normalizing AP-1 activity to modulate hormonal output and redox homeostasis, providing a translational path toward targeted interventions.

In conclusion, by integrating multi-omics and functional assays, this study identifies FOS and JUN as key mediators of adrenal redox-steroidogenic crosstalk. Localized AP-1 activation in non-neoplastic adrenal cortical regions functions as a redox-sensitive protective mechanism, whereas APAs bypass this checkpoint through intrinsic antioxidant adaptations. These findings highlight a role for oxidative stress in regulating adrenal steroidogenesis. Future investigations into MAPK-AP-1 signaling dynamics may not only refine our understanding of adrenal tumor biology but also open new avenues for targeted therapeutic strategies to mitigate hormone excess.

## Funding

This research was funded by the 10.13039/501100001659Deutsche Forschungsgemeinschaft (10.13039/501100001659DFG) project number 444776998 to TAW (WI 5359/2-1) and MR (RE 752/31-1) and project number 314061271-TRR 205 “The Adrenal: Central Relay in Health and Disease” to TAW and MR. JW is supported by the China Scholarship Council.

## CRediT authorship contribution statement

**Jia Wei:** Conceptualization, Data curation, Formal analysis, Investigation, Methodology, Writing – original draft. **Eleonora Duregon:** Investigation, Methodology, Writing – review & editing. **Mauro G. Papotti:** Investigation, Methodology, Writing – review & editing. **Thomas Knösel:** Investigation, Methodology, Writing – review & editing. **Martin Bidlingmaier:** Investigation, Writing – review & editing. **Silviu Sbiera:** Investigation, Methodology, Writing – review & editing. **Martin Reincke:** Funding acquisition, Project administration, Supervision, Writing – review & editing. **Tracy Ann Williams:** Conceptualization, Data curation, Funding acquisition, Project administration, Resources, Supervision, Writing – original draft.

## Declaration of competing interest

The authors declare that they have no known competing financial interests or personal relationships that could have appeared to influence the work reported in this paper.

## Data Availability

Data will be made available on request.
